# Exploring New and Promising Genetic Biomarkers for Evaluating Traumatic Brain Injuries: A Case-Control Study

**DOI:** 10.1007/s11064-024-04292-9

**Published:** 2024-12-06

**Authors:** Yasmin Kamal Abd Rabou, Abeer Ahmed Zayed, Sally A. Fahim, Marwa Abdelgwad, Ahmed El Fiki, Nermin Nabil Fayed

**Affiliations:** 1 Department of Forensic Medicine and Clinical Toxicology, Faculty of Medicine, Cairo University, Kasr Alainy Street, Cairo, 11562 Egypt; 2 Department of Biochemistry, School of Pharmacy, New Giza University (NGU), New Giza, Km 22 Cairo- Alexandria Desert Road, P.O. Box 12577, Giza, Egypt; 3 Department of Biochemistry, Faculty of Medicine, Cairo University, Kasr Alainy Street, Cairo, 11562 Egypt; 4 Department of Neurosurgery, Faculty of Medicine, Cairo University, Kasr Alainy Street, Cairo, 11562 Egypt

**Keywords:** hsa-miR-16-5p, MALAT 1, GSK-3β, AKT3, TBI

## Abstract

Traumatic brain injury (TBI) is a common cause of morbidity and death in all age groups, with an estimated 50 million people having brain injury due to trauma each year. Accurate blood-based biomarkers are needed to assist with diagnosis of patients across the spectrum of time and severity. Our objectives were to explore the diagnostic precision of time- and severity- related four blood-based biomarkers: AKT3, GSK-3β, hsa-miR-16-5p, and MALAT-1 for TBI for the purpose of diagnosis, prognosis, and follow-up. 40 samples were recruited as the following: 30 TBI patients and 10 healthy volunteers as controls with matched age and sex. They were divided according to the Glasgow Coma Scale into mild (mTBI), moderate (modTBI), and severe(sTBI) TBI. Blood samples were withdrawn at entry, and after 5 and 30 days, RT-PCR was used for measuring the expression level. The results showed upregulated expression levels of AKT3, hsa-miR-16-5p and significantly downregulated expression levels of GSK-3β in TBI patients compared to controls at all timings measured. mTBI patients showed a higher expression level of hsa-miR-16-5p compared with modTBI, and sTBI patients. MALAT-1 level showed a significant increase in severe cases only. We concluded that AKT3, hsa-miR-16-5p, and GSK-3β are excellent diagnostic biomarkers in TBI patients at initial assessment, as well as at 5 and 30 days following the injury. Moreover, MALAT-1 had good diagnostic value in sTBI patients, and its prognostic value extends to 30 days. GSK-3β was an excellent biomarker for detecting mTBI.

## Introduction

Traumatic brain injury (TBI), often referred to as the “silent epidemic”, refers to the functional impairment of the brain caused by external forces [1]. TBI is represents the most significant contributor to death and disability globally among all trauma-related injuries, with an estimated 27.16 million new cases and 48.99 million existing cases [2–4].

The Glasgow Coma Scale (GCS), a longstanding clinical tool dependent on physical examination findings, is utilized to measure the severity of TBI [5]. Predicting the outcome of patients after a TBI lacks precision when relying exclusively on clinical signs and radiographic observations. Individuals with apparently similar injuries often experience markedly different outcomes [6]. TBI can give rise to various neurological and behavioral disorders, making it challenging to evaluate prognosis and monitor associated injuries arising from secondary inflammatory processes [7].

Many of the studied biomarkers demonstrate low sensitivity, particularly in cases of Mild Traumatic Brain Injury (mTBI) where individuals lack visible intracranial lesions and exhibit minimal observable symptoms. Additionally, the timing of the injury can have significant relevance in forensic investigations as it can influence the stage of injury progression and healing processes. Moreover, the biomarkers associated with inflammation, tissue damage, and recovery pathways vary depending on the time elapsed since the injury occurred [8].

Non-coding RNAs (ncRNAs) have emerged as key regulators in various biological processes, as evidenced by multiple research studies preventing neuronal damage resulting from brain injuries, potentially leading to better outcomes for individuals with TBI [9–13]. Moreover, in recent decades, there has been substantial progress in the advancement of high-throughput RNA sequencing and bioinformatics and tools to study how genes are activated or inhibited, showing significant growth in this field [14–16].

MicroRNAs (miRNAs) are types of regulatory non-coding RNAs. These are important in managing post-transcriptional gene expression across various species by aiming multiple mRNAs through sequence complementarity and typically suppressing these target RNAs [17]. Dysregulation of miRNAs has a role in neurodevelopment and the proper functioning of the brain by regulating vital cellular processes related to both neuronal injury and their subsequent repair mechanisms [18]. Lower levels of hsa-miR-16-5p have been attributed to faster fracture healing in people with TBI. This effect is achieved by stimulating the growth of osteoblasts and suppressing apoptosis [19].

Long non-coding RNAs (LncRNAs) represent a relatively new class of RNA molecules, typically exceeding 200 nucleotides in length [10]. Among these LncRNAs, MALAT-1 is noteworthy for its influence on endothelial cell function and the growth of blood vessels [20]. Increased expression of MALAT-1 has been linked to improvements in brain edema in cases of TBI [21].

Glycogen Synthase Kinase 3β (GSK-3β) was known as a controller of glycogen metabolism and was linked to a diverse array of cellular processes, encompassing protein synthesis, cell growth, cellular differentiation, cell motility, and the control of apoptosis. It is worth noting that neuronal cell derangement especially apoptosis has been linked to GSK-3β activity [22]. It was found that inhibition of GSK-3β prevented cognitive deficits after TBI [23]. Akt1, Akt2, and Akt3 are three forms of the AKT family, known as protein kinase b. Akt3 is the predominant form of AKT found in the brain, and it has been reported that the phosphorylation of Akt is also implicated in the signaling pathways that influence cell survival following TBI [24].

Therefore, the aim of our study is to detect the levels of MALAT-1, hsa-miR16-5p, GSK-3β, and AKT3 in the plasma of mild, moderate, and severe TBI (mTBI, ModTBI, sTBI) patients after 5 and 30 days of trauma and compare them to the baseline at injury day. Therefore, exploring their potential utility as biomarkers for diagnosing, prognosing, and monitoring TBI patients, in addition to their significant relevance in forensic investigation. Moreover, the target genes and functional analysis has been identified using bioinformatics.

## Subjects and Methods

### Bioinformatics and Rationale of Biomarkers Choice

GSK-3β and AKT3, relevant protein-coding genes, were selected from the Gene Atlas (http://genatlas.medecine.univ-paris5.fr/) as they are well expressed in the TBI patients [25] and were involved in cognitive deficits and cell survival after TBI [23, 24]. Then, hsa-miR-16-5p was identified by TargetScan (https://www.targetscan.org/vert_80/) [26], miRDB (https://mirdb.org/) [27], and RNAv22 (https://cm.jefferson.edu/rna22/Interactive/) [28] targeting both GSK-3β and AKT3, in addition to its role in neurodevelopment, the proper functioning and repair mechanisms of the brain following TBI [18, 19]. Finally, the lncRNA, MALAT-1 is determined by accessing the Diana database (http://carolina.imis.athenainnovation.gr/diana_tools/web/index.php?r=tarbasev8%2Findex) [29] and confirming its role in both sponging hsa-miR-16-5p and improving brain edema in cases of TBI [21]. Cytoscape software (V 3.10.1) was then used to form networks illustrating the miRNA target genes [30]. DAVID database (https://david.ncifcrf.gov/summary.jsp) [31] was used for gene ontology to categorize what they do, where they work in the cell, and how they contribute to cell function., in addition to functional enrichment analysis including the KEGG pathway.

### Study Design

**Fig. 1:**
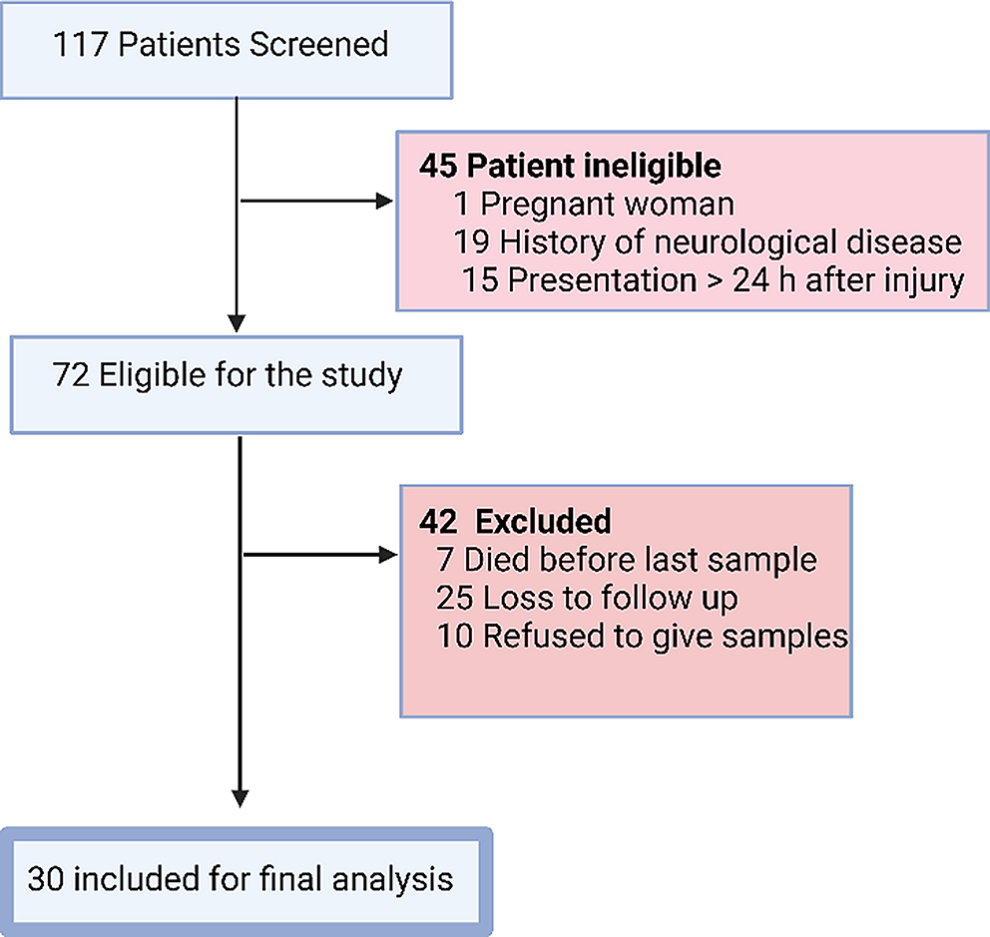
Fow chart of participants

This case-control study comprises 30 TBI cases and 10 healthy controls, TBI cases were recruited from the neurosurgery, emergency departments, and outpatient clinic at Kasr Alainy Hospital, Cairo University, between July 2022 and January 2023 (Fig. 1). This study was conducted with ethical approval at Kasr Alainy Hospital (MD-109-2021) and conducted according to the ethical principles outlined in the Declaration of Helsinki. All participants provided written informed consent. Participants with TBI either provided written informed consent or, in cases where they were incapable or unconscious of providing consent, approval was sought from their legal representative. Patients who entered the hospital without caregivers or guardians are excluded from the study.

TBI cases were differentiated according to GCS, into mTBI (13–15) scores, modTBI (9–12) scores, or sTBI (3–8) scores [32]. Additionally, 10 healthy controls matched for age- and sex-seeking routine health check-ups were recruited from the same hospital. Every patient in our research received a CT scan within 24 h upon arrival at the emergency department.

The inclusion criteria for patients included being adults between the ages of 18 and 45, irrespective of gender, and having experienced recent head trauma. Exclusion criteria encompassed pregnant or nursing women, individuals with diabetes, hypertension, or other significant cardiovascular diseases, and those with debilitating neurological conditions of pathological origin, concurrent spinal cord injuries, or injuries resulting from substance abuse or alcoholism.

### Sample Collection and Biochemical Analysis

Five milliliters of blood were collected from both cases and control groups in EDTA tubes within 24 h of admission then sent to the laboratory within 30 min of collection. The blood was then centrifuged at 4,000 xg for 10 min to isolate the plasma and immediately frozen at − 80^o^C for subsequent RNA extraction. To minimize bias, group allocation (intervention vs. control) and outcome assessment were blinded.

#### RNA Extraction

Total RNA was extracted from the plasma samples using a commercially available kit using a commercially available kit (miRNeasy, Qiagen) designed specifically for extracting small RNAs. The concentration and purity of the extracted RNA were quantified using a NanoDrop spectrophotometer. ^®^ (ND)-1000 spectrophotometer (NanoDrop Technologies, Wilmington, DE, USA.

#### Reverse Transcription (RT) and real-time Quantitative PCR (qPCR)

- **LncRNA and Gene Expression**: ELK Green One-Step qRT-PCR Super Mix kit (Cat. No. Equations 007-02) was used to quantify the expression of the lncRNA; MALAT-1, and GSK-3β and AKT3 genes. This kit utilizes a three-step cycling protocol:
  - ○ Reverse transcription: 1 cycle at 50 °C for 15 min.
  - ○ Predenaturation: 1 cycle at 95 °C for 2 min.
  - ○ Amplification: 40 cycles of:
    - Denaturation: 95 °C for 10 s.
    - Annealing: 50–60 °C for 30 s.
    - Extension: 72 °C for 30 s.
- **MicroRNA Expression**: For microRNA-16-5p (hsa-miR-16-5p) quantification, a separate miRCURY LNA RT Kit (Qiagen, Cat. No. 339345) was used for reverse transcription. Two microliters (2 µL) of extracted RNA were reverse transcribed into cDNA in a final reaction volume of 10 µL. The RT reaction consisted of incubation at 37 °C for 60 min, followed by enzyme inactivation at 85 °C for 5 s.
- **Real-Time PCR Setup**: For both lncRNA/gene and miRNA analysis, a 10 µL reaction volume per well was prepared. The real-time PCR program used the following steps:
  - ○ Initial activation: 95 °C for 2 min.
  - ○ Cycling:
    - Denaturation: 95 °C for 10 s.
    - Combined annealing and extension: 56 °C for 60 s (40 cycles).

Gene expression levels of MALAT-1, GSK-3β, and AKT3 were measured using quantitative reverse transcription PCR (RT-qPCR) on an Applied Biosystems StepOne™ instrument (version 3.1, USA). GAPDH was used as a reference gene to normalize the expression of these genes. For the hsa-miR-16-5p, U6 was used as the reference gene. The 2^-ΔΔCt^ method was employed to analyze the RT-qPCR results.

All Primers are listed in Table 1 and their specificity was checked using primer Blast (https://www.ncbi.nlm.nih.gov/tools/primer-blast/). All the primers utilized demonstrated 100% efficiency. To assess the efficiency, a 10-fold dilution of our target was prepared, and a regular real-time PCR run was conducted. The Ct (threshold cycle) values obtained from the qRT-PCR experiment were plotted on the y-axis (logarithmic scale) against the corresponding known concentrations of the starting material (e.g., RNA) on the x-axis using GraphPad software, A linear regression analysis was performed on the plotted data points to generate a best-fit trend line. The slope of the generated trend line was then calculated. Finally, the PCR efficiency (E) was determined using the equation: E = -1 +10^(-1/slope).

**Table 1:** List of primers

	Primer	Accession number
hsa-miR-16-5p	F-3’TGGGGTAGCAGCACGTAAA’5 R-3’CTCAACTGGTGTCGTGGAGTC’5	MIMAT0000069
MALAT-1	F-3’GAATTGCGTCATTTAAAGCCTA’5 R-3’GTT GTTTCATCCTACCACTCCCAATTAAT’5	NR_145459.1
GSK3-β	F-3’GGAACTCCAACAAGGGAGCA’5 R-3’TTCGGGGTCGGAAGACCTTA’5	NM_001146156.2
Akt3	F-3’TGGACCACTGTTATAGAGAGAACATTT’5 R-3’TGGATAGCTTCCGTCCACTC’5	NM_001206729.2
GAPDH	F-3’ACAGTCAGCCGCATCTTCTT’5 R-3’GACAAGCTTC CCGTTCTCAG’5	NM_001357943.2
U6	F-3’CTCGCTTCGGCAGCACA’5 R-3’AACGCTTCACGAATTTGCGT’5	NR_104084.1

MALAT-1: metastasis-associated lung adenocarcinoma transcript 1, GSK-3β: Glycogen synthase kinase B, Akt3: AKT serine/threonine kinase 3,GAPDH: Glyceraldehyde-3-phosphate dehydrogenase, U6:urokinase

### Statistical Analysis

The data underwent encoding and entry into the statistical software program called SPSS V28 (IBM Corp. in Armonk, NY, USA). For numerical data, data was represented using mean and standard deviation. Categorical data was represented as number and percent. unpaired t-tests or analysis of variance (ANOVA) were employed to compare between groups, for normally distributed quantitative variables. Conversely, non-parametric tests such as the Kruskal-Wallis and Mann-Whitney tests were utilized for quantitative variables that did not follow a normal distribution [33], For categorical data analysis, the Chi-squared (χ2) test was conducted, and in cases where the expected frequency was less than five, the Fisher-exact test was utilized [33]. The correlation between quantitative variables was assessed through the computation of Spearman’s correlation coefficient [34]. To identify the optimal cutoff values for significant markers in detecting cases, ROC curves were created.

## Results

### Demographic and Medicolegal data of TBI Patients

Table 2 shows the characteristics of the study participants. Patients were older than 18 years, Age and gender distribution were comparable between the groups (no significant differences (*P* > 0.05). The patients were subdivided based on severity: mTBI, modTBI, and sTBI according to their GCS scores with a median of 15,12, and 6, respectively. 100% of the severely injured patients had hemorrhage of which 40% died. homicidal is equal to abuse and deliberate assault and violence as hitting by a brick or a stick intentionally, deliberate throwing the case on stairs in quarrel or gun shot.

**Table 2:** Demographic and injury characteristics of study participants

	TBI (*n* = 30)			*P*-Value
	Mild (*n* = 10)	Moderate (*n* = 10)	Severe (*n* = 10)	
**Age**	30.1 ± 11.4	31 ± 8.9	29.9 ± 8.2	0.68
**Sex**				
Male Female	9 (90%) 1 (10%)	9 (90%) 1 (10%)	9 (90%) 1 (10%)	0.19
**Occupation**				
Manual work Mental work Student None	7 (70%) 0 (0%) 1 (10%) 2(20%)	5 (50%) 3 (30%) 0 (0%) 2 (20%)	6 (60%) 1 (10%) 2 (20%) 1 (10%)	0.39
**Residence**				
Urban Rural	5 (50%) 5 (50%)	5 (50%) 5 (50%)	7 (70%) 3 (30%)	0.58
**Mechanism of Injury**				
Road traffic injury Fall from height Violence Work accident Other	2 (20%) 0 (0%) 5 (50%) 3 (30%) 0 (0%)	3 (30%) 2 (20%) 4 (40%) 0 (0%) 1 (10%)	7 (70%) 1 (10%) 0 (0%) 0 (0%) 2 (20%)	0.02*
**Manner of injury**				
Accidental Homicidal	5 (50%) 5 (50%)	6 (60%) 4 (40%)	8 (80%) 2 (20%)	0.36
**GCS**	15 (13–15)	12 (9–12)	6 (3–8)	< 0.0001****
**CT changes**				
Contusion Hemorrhage Extradural Subdural Intracerebral Subarachnoid Combined DAI	5 (50%) 5 (50%) 4 0 0 1 0 0 (0%)	0 (0%) 8 (80%) 3 2 2 1 2 (20%) 0 (0%)	0 (0%) 3 (30%) 1 0 1 1 4 (40%) 3 (30%)	< 0.001***
**Outcome**				
Recovered Complicated Permanent infirmity Died	10 (100%) 0 (0%) 0 (0%) 0 (0%)	7 (70%) 1 (10%) 2(20%) 0 (0%)	0 (0%) 6 (60%) 0 (0%) 4 (40%)	< 0.0001****
**Headache**				
Yes No	9 (90%) 1 (10%)	9 (90%) 1 (10%)	NA	1
**Vertigo**				
Yes No	2 (20%) 8 (80%)	5 (50%) 5 (50%)	NA	0.16
**Insomnia**				
Yes No	2 (20%) 8 (80%)	2 (20%) 8 (80%)	NA	1
**Cognitive**				
Yes No	1 (10%) 9 (90%)	4 (40%) 6 (60%)	NA	0.12
**Time od injury to admission**	6.7 ± 2.04	5.15 ± 2.12	4.8 ± 2.33	0.806

* Significant at *p* < 0.05, ** significant at *p* < 0.01, *** significant at *p* < 0.001, **** significant at *p* < 0.0001. Data are expressed as mean ± SD, or n (%). All Data was analyzed using Chi square (X^2^) test, while age was analyzed using one-way ANOVA and Tukey’s multiple comparisons test. GCS: Glasgow coma scale, DAI: diffuse axonal injury

### AKT3 and GSK-β3 gene Expression Levels Between Different TBI Groups and at Different Times (0, 5, and 30 days)

**Fig. 2:**
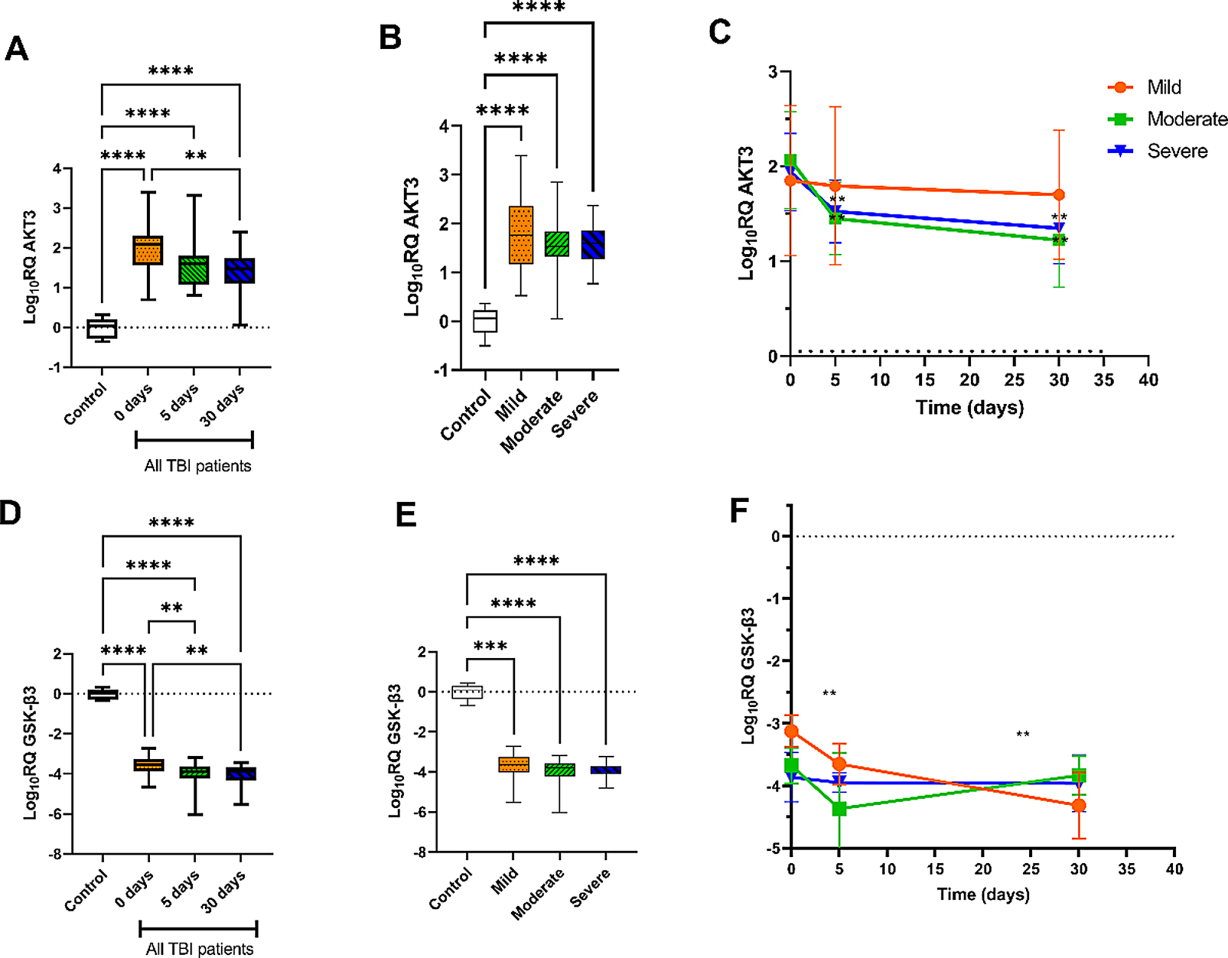
The gene expression level of AKT3 and GSK-β3 in all TBI patients at 0, 5, and 30 days (**A** & **D**), in mTBI, modTBI, and sTBI patients (**B** & **E**), and in mTBI, modTBI, and sTBI patients at 0, 5, and 30 days following the trauma (**C** & **F**), data are shown as log10RQ. Error bars show the standard deviation. A p-value < 0.05 was considered statistically significant, * significant at *p* < 0.05, ** significant at *p* < 0.01, *** significant at *p* < 0.001, ****significant at *p* < 0.0001

The results showed a significant increase AKT3 levels in TBI patients compared to the control at 0, 5, and 30 days (Fig. 2A). Moreover, Fig. 2B shows that AKT3 expression level was significantly increased in mTBI, modTBI, and sTBI cases in comparison to the control at P-value < 0.001, while no significant change was detected according to the severity of the TBI. When comparing the AKT3 levels among the three time points, it didn’t change in patients with mTBI, while its level showed a significant decrease in modTBI and sTBI patients after 5 and 30 days (*P* < 0.01, Fig. 2C) when compared to the baseline time. On the other hand, GSK-3β expression level decreased in TBI patients compared to the control at 0, 5, and 30 days. The decrease in GSK-3β expression level was gradual over time where its level decreased after 5 and 30 days compared to its level at day zero (Fig. 2D). GSK-3β level was significantly decreased in all TBI severity groups in comparison to the control (Fig. 2E). Moreover, GSK-β3 levels further decreased after 5 and 30 days in mTBI cases compared to T0, while their levels didn’t significantly change after 5 and 30 days in modTBI and sTBI patients (Fig. 2F).

### The Expression Level of the miRNA; hsa-miR-16-5p and the lncRNA; MALAT-1 of Different TBI Severity Patients at Different Timings

**Fig. 3:**
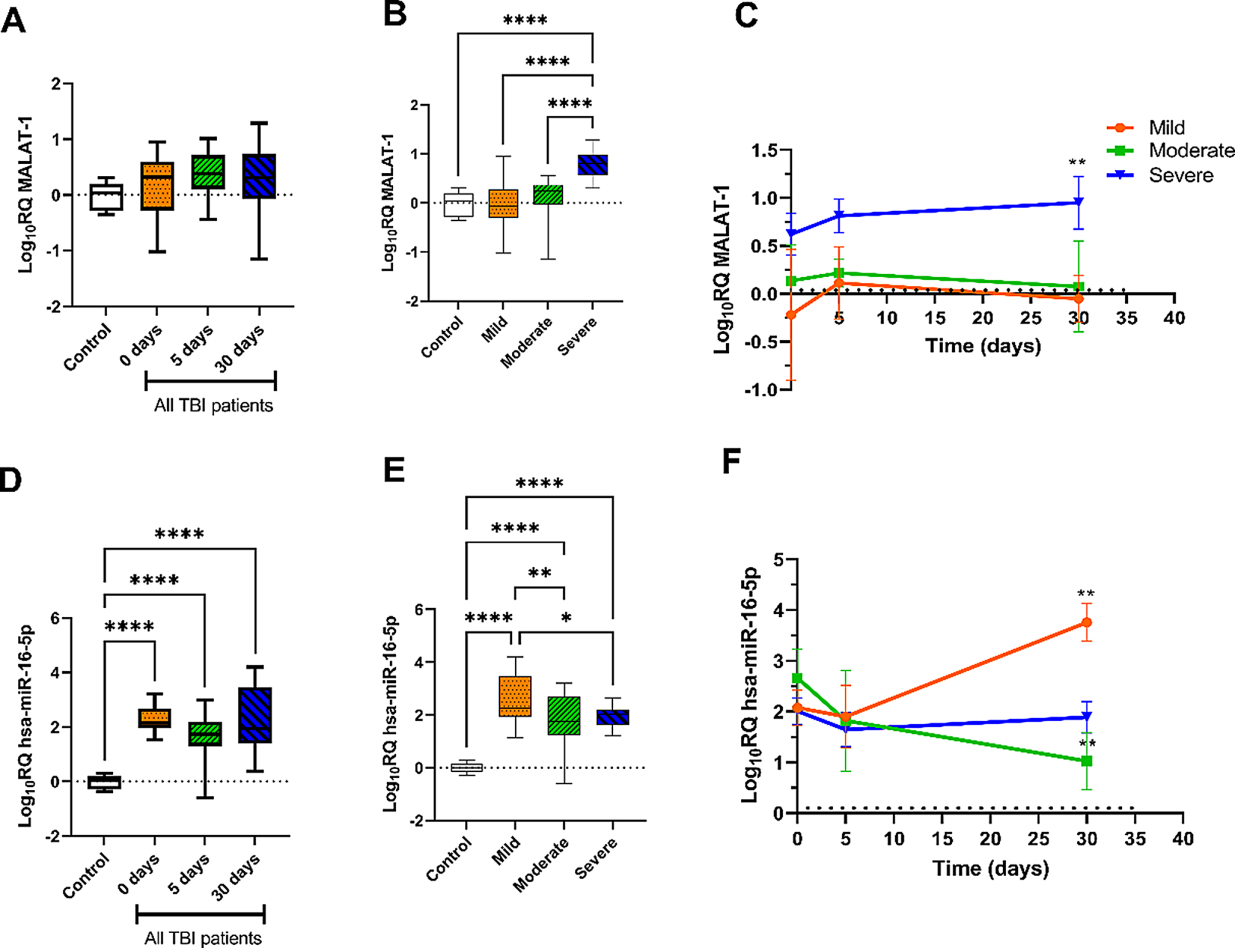
MALAT-1 and hsa-miR-16-5p expression level in all TBI patients at 0, 5, and 30 days (**A** & **D**), in mTBI, modTBI, and sTBI patients (**B** & **E**), in mTBI, modTBI, and sTBI patients at 0, 5, and 30 days following the trauma (**C** & **F**). Data are represented as log_10_RQ. Error bars represent the standard deviation. A p-value < 0.05 was considered statistically significant, ^*^ significant at *p* < 0.05, ^**^ significant at *p* < 0.01, ^***^ significant at *p* < 0.001, ^****^ significant at *p* < 0.0001

MALAT-1 level showed a significant increase in severe cases in comparison to the control, mTBI and modTBI, cases (*P* < 0.0001), while its level did not significantly change in individuals with mTBI or modTBI compared to a healthy control group (Fig. 3B). When comparing the MALAT-1 levels among the three time points, the change wasn’t significant even after 30 days in patients with mTBI and modTBI, while its level showed a significant increase after 30 days in sTBI patients (*P* < 0.01, Fig. 3C) when compared to the baseline time. The results showed a significant increase in hsa-miR-16-p levels in TBI patients compared to the control group at 0, 5, and 30 days (*P* < 0.0001) as shown in Fig. 3D. hsa-miR-16-5p level increased in all TBI cases compared to the control (*P* < 0.0001), interestingly, mTBI patients showed a higher expression level compared with modTBI, and sTBI patients with *P* < 0.01 and < 0.05, respectively (Fig. 3E). Moreover, after 30 days following trauma, mTBI patients, in contrast to modTBI, showed an increase in the level of hsa-miR-16-5p compared to the baseline time (*P* < 0.0001, Fig. 3F).

### Diagnostic Performance of the MALAT-1, hsa-miR-16-5p, GSK-3β, and AKT3

ROC curve results indicated that AKT3, GSK-3β, and hsa-miR-16-5p were excellent diagnostic biomarkers for differentiation between TBI cases and control at time of admission and even after 5 and 30 days with area under the curve (AUC) exceeding 0.95 at all timings (*P* < 0.0001, Table 3). Furthermore, the levels of various biomarkers could distinguish between different severity outcomes of TBI based on the timing of assessment. Specifically, GSK-3β could differentiate between mild TBI and other cases at 0 time and after 5 days, whereas MALAT-1 after 30 days post-injury. On the other hand, MALAT-1 could discriminate between sTBI from mTBI and modTBI at 0 time (AUC=0.85, sensitivity=90% and specificity=70%, *P*=0.002) and after 5 (AUC=0.985, sensitivity=90 and specificity=95, *P*<0.0001) and 30 days of injury with AUC of 0.995, sensitivity and specificity equal 90% and 95%, respectively at *P*<0.0001.

**Table 3:** ROC curve analysis of MALAT-1, hsa-miR-16-5p, GSK-3β, and AKT3 stratified by time and severity

Time (days)	Biomarkers	AUC	95% CI	*P*-Value	Sensitivity (%)	Specificity (%)
Control vs. all TBI patients						
0	AKT3	**1**	**1–1**	< 0.0001****	**100**	**100**
	GSK-3β	**1**	**1–1**	< 0.0001****	**100**	**100**
	MALAT-1	0.65	0.49–0.815	0.15	56.7	70
	hsa-miR-16-5p	**1**	**1–1**	< 0.0001****	**100**	**100**
5	AKT3	**1**	**1–1**	< 0.0001****	**100**	**100**
	GSK-3β	**1**	**1–1**	< 0.0001****	**100**	**100**
	MALAT-1	0.78	0.637–0.923	0.009**	76	70
	hsa-miR-16-5p	**0.967**	**0.92-1**	< 0.0001****	**96**	**100**
30	AKT3	**0.983**	**0.948-1**	< 0.0001****	**96.7**	**100**
	GSK-3β	**1**	**1–1**	< 0.0001****	**100**	**100**
	MALAT-1	0.71	0.551–0.869	0.049	66.7	70
	hsa-miR-16-5p	**1**	**1–1**	< 0.0001****	**100**	**100**
**mTBI vs. modTBI and sTBI**						
0	AKT3	0.58	0.34–0.81	0.48	60	70
	GSK-3β	**0.94**	**0.86-1**	< 0.0001****	**80**	**90**
	MALAT-1	0.76	0.54–0.99	0.02*	75	80
	hsa-miR-16-5p	0.64	0.44–0.84	0.22	65	60
5	AKT3	0.39	0.135–0.655	0.35	70	40
	GSK-3β	**0.8**	**0.619–0.981**	0.008**	**80**	**80**
	MALAT-1	0.78	0.599–0.9961	0.01*	85	60
	hsa-miR-16-5p	0.48	0.24–0.72	0.89	60	30
30	AKT3	0.31	0.078–0.542	0.09	60	30
	GSK-3β	0.235	0.067–0.403	0.02*	90	65
	MALAT-1	**0.87**	**0.74–0.99**	0.001***	**90**	**75**
	hsa-miR-16-5p	1	1–1	< 0.0001****	100	100
**sTBI vs. mTBI and modTBI**						
0	AKT3	0.49	0.27–0.72	0.93	60	40
	GSK-3β	0.79	0.624–0.96	0.009**	80	75
	MALAT-1	**0.85**	**0.72–0.99**	0.002**	**90**	**70**
	hsa-miR-16-5p	0.31	0.122–0.498	0.09	60	35
5	AKT3	0.505	0.29–0.714	0.96	70	50
	GSK-3β	0.6	0.396–0.804	0.37	70	60
	MALAT-1	**0.985**	**0.953-1**	< 0.0001****	**90**	**95**
	hsa-miR-16-5p	0.365	0.167–0.563	0.23	70	25
30	AKT3	0.45	0.23–0.668	0.66	60	35
	GSK-3β	0.425	0.13–0.667	0.509	60	25
	MALAT-1	**0.995**	**0.97-1**	< 0.0001****	**90**	**95**
	hsa-miR-16-5p	0.455	0.246–0.664	0.69	60	45

Bolded result indicates AUC at least 0.8 (at least ‘good’ discrimination)

* Significant at *p* < 0.05, ** significant at *p* < 0.01, *** significant at *p* < 0.001, **** significant at *p* < 0.001. ROC, Receiver–operator curves

### Prognostic Performance of the MALAT-1, hsa-miR-16-5p, GSK-3β, and AKT3

**Fig. 4:**
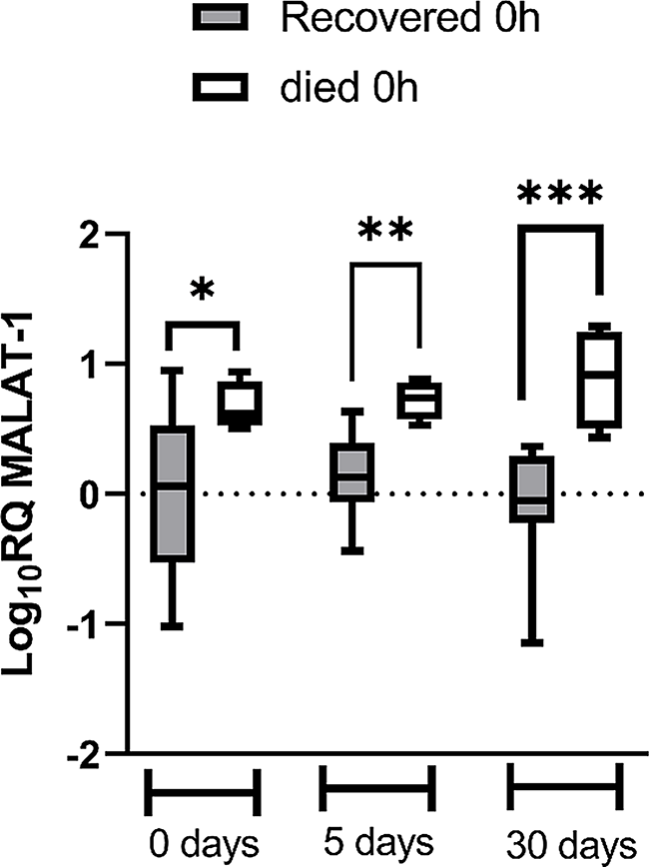
The expression level of MALAT-1 according to patients’ outcome

Regarding prognostic significance of these biomarkers, the outcomes were evaluated, with some patients experiencing recovery, some encountering complications, and others died. As shown in Fig. 4, MALAT-1 level significantly increased in patients who died in comparison to those who recovered. This increase was obvious when it was measured at 0, 5 or 30 days at *p* < 0.05, 0.01, 0.001, respectively. The other biomarkers didn’t show significant change according to the outcome of patients.

### Correlation Between Different Biomarkers and GCS

**Fig. 5:**
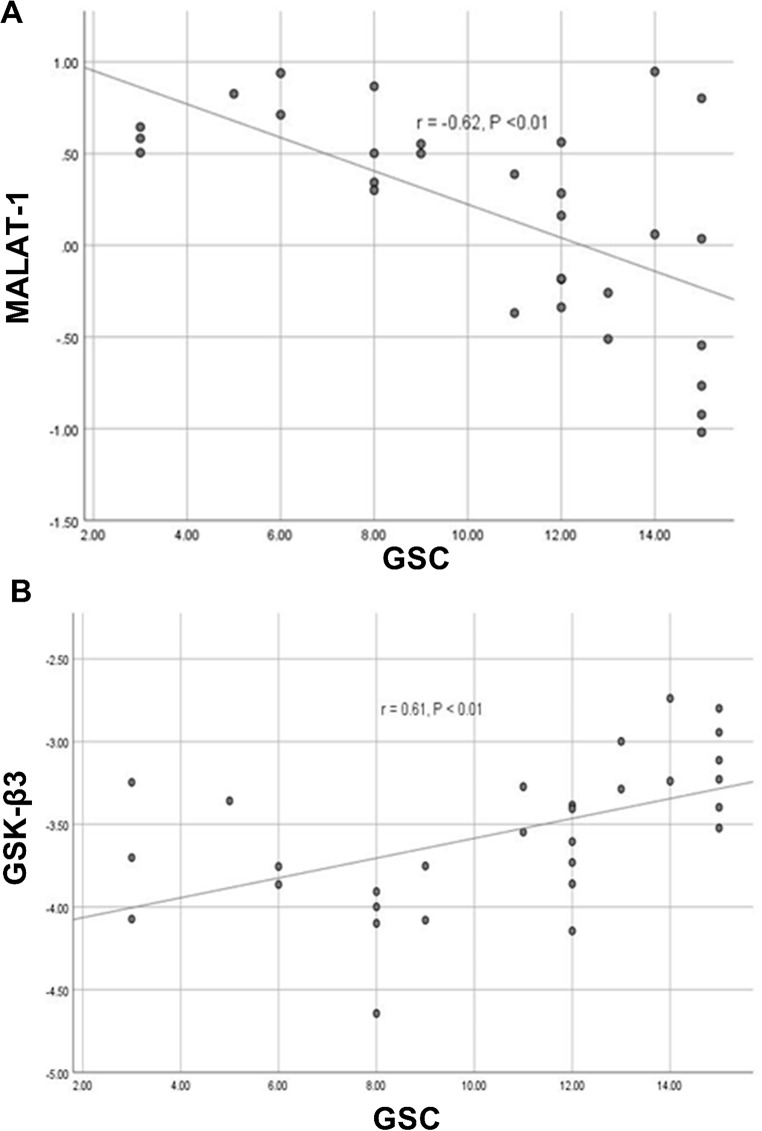
Correlation between MALAT-1 (**A**) and GSK-3β (**B**) with GCS

Our analysis revealed opposite trends between gene expression and GCS scores. MALAT-1 levels showed a strong negative correlation (*r* = -0.62, *P* < 0.01) with GCS, meaning higher MALAT-1 levels were associated with lower GCS scores (Fig. 5A). Conversely, GSK-3β displayed a strong positive correlation (*r* = 0.61, *P* < 0.001) with GCS (Fig. 5B).

### Integrated Targets Prediction of MALAT-1 and hsa-miR-166-5a Target Genes and their Functional Enrichment Analysis and GO Annotation

**Fig. 6:**
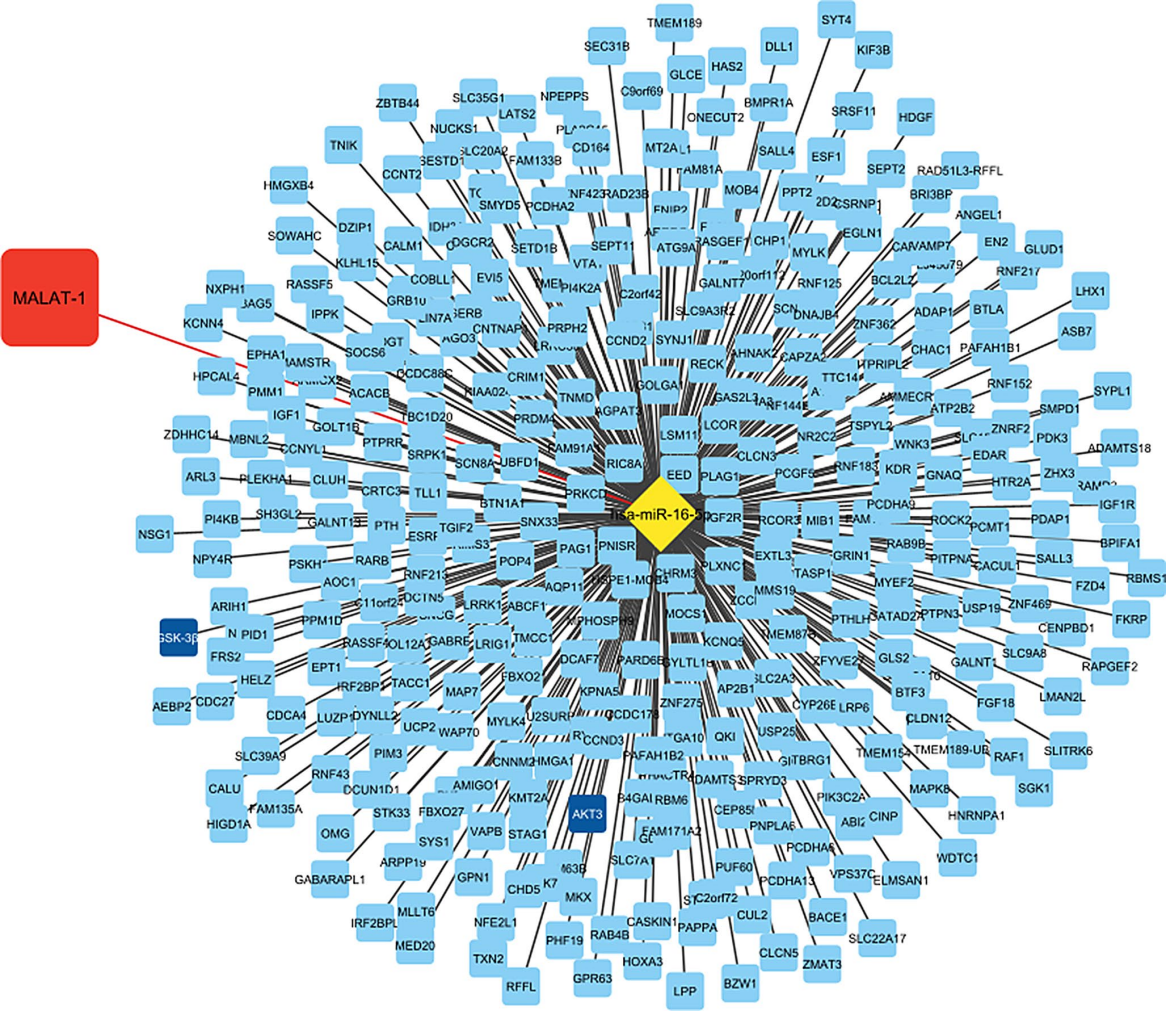
The lncRNA-miRNA-mRNA interaction network constructed with cytoscape. The network consists of the lncRNA MALAT-1 (red node), has-miR-16-5p (yellow), and their target genes (blue)

LncRNA was reported to sponge miRNAs which in turn regulates gene expression by attaching to a specific 3’UTR of target genes and hindering their translation into proteins. hsa-miR-16-5 was one of the targeted miRNAs of the lncRNA MALAT-1. To understand the core function of hsa-miR-16-5a, its target genes were identified. A total of 361 overlapping genes were predicted using different prediction tools: TargetScan, RNAv22 and miRDB (Fig. 6).

**Fig. 7:**
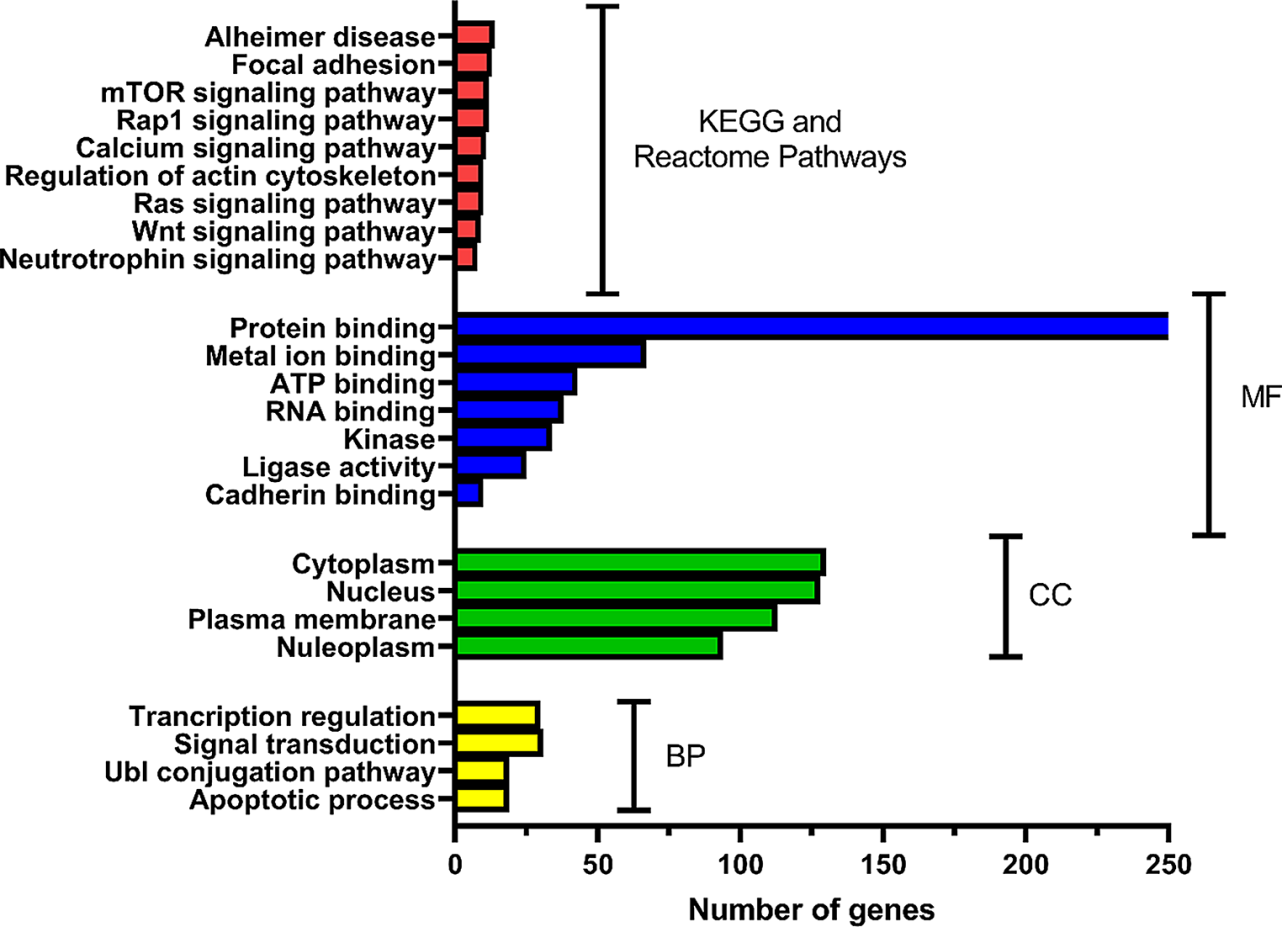
Gene ontology analysis for miR-16a-5p. Bar graphs show the enriched GO terms of molecular function (MF), cellular components (CC), and biological process (BP) arranged according to the number of genes (n) involved. Results are shown only for P value match of < 0.001 and *n* ≥ 5

To get more insight into the cellular roles and patterns of regulation of hsa-miR-16-5p, the 361 overlapping predicted target genes were assessed by using the functional enrichment tool David and ShinyGO. The target genes were involved in various molecular functions (MFs) activities such as protein, metals, ATP, RNA, and cadherin binding, in addition to their involvement in ligase activity. When we analyzed the functions of these 361 target genes using Gene Ontology (GO) biological processes (BPs), we found they were primarily involved in several key processes, including transcription regulation, signal transduction, protein modification through ubiquitination (Ubl conjugation), and cell death pathways (apoptotic pathway). The molecules targeted by these circRNAs were mainly found in specific parts of the cells, the cytoplasm, nucleus, and plasma membrane. Moreover, the results showed that in the KEGG functional sets, mTOR, Rap1, calcium, Ras, Wnt signalling pathways were the common pathways enriched. Moreover, these target genes were mainly involved in Alzheimer’s disease (Fig. 7).

## Discussion

Novel biomarkers for TBI are crucial for diagnosing, prognosing, and monitoring TBI patients, in addition to their significant relevance in forensic investigation due to their potential for fatalities, even when there are limited observable symptoms. Additionally, the timing of the injury can have significant relevance in forensic investigations as it can influence the stage of injury progression and healing processes [8]. Moreover, the biomarkers associated with inflammation, tissue damage, and recovery pathways vary depending on the time elapsed since the injury occurred. Nonetheless, the complicated molecular mechanisms underlying traumatic processes and their effect on the brain remain largely unknown. The prospect of discovering novel biomarkers in the blood that can predict the severity and timing of an injury presents an exciting frontier in the field of forensic medicine.

Recent systematic reviews have shown that a combination of tests involving the immediate identification of certain ncRNAs and mRNAs, which are either upregulated or downregulated, in various biological fluids, including CSF, blood or serum, can be effectively employed in clinical settings for nonstandard assessments and the prediction of TBI severity [35].

In our current research, AKT3 was identified as an excellent diagnostic biomarker, showing a significant increase in all TBI cases but unable to distinguish between TBI severities. Although AKT3 levels are influenced by time and there was a notable decrease in AKT3 levels after 5 days, reaching their lowest point after 30 days in cases of modTBI and sTBI, but it can still be utilized as a biomarker for TBI patients at the initial assessment or after 5- or 30-days post injury. This finding aligns with prior research that reported the degradation of Akt3 within just one hour following a stroke event [36]. Moreover, in the study of Zhao et al., (2012), AKT3 levels in rodents were increased after TBI, at 24 h post-injury then decreased after 3 days and reached their lowest point after 7 days [37]. AKT3 was found to provide robust neuronal protection by enhancing mTOR activity [38]. Moreover, AKT3 has been demonstrated to regulate the activity of GSK-3β through phosphorylation in response to the binding of growth factors via AKT3. This phosphorylation leads to the inactivation of GSK-3β and is believed to contribute to the anti-apoptotic properties associated with the activation of AKT3, thereby playing a protective role in TBI [39].

The present study marks a pioneering attempt to investigate the expression level of GSK-3β in varying levels of severity of TBIs at three distinct time intervals. In our study, GSK-3β was identified as a diagnostic biomarker not only capable of distinguishing between TBI patients and the control group at different timing but also between patients with mTBI, modTBI, and sTBI. In addition, GSK-3β levels showed a further decrease after 5 and 30 days in mild TBI cases. Dash et al. (2011) [39] reported that phosphorylation and inactivation of GSK-3β increases after injury and became statistically significant by day 3. Moreover, Shapira et al. [40] reported the role of GSK-3 in early depressive behavior induced by mTBI. The AKT/GSK-3β/CRMP-2 signaling cascade is known to play a role in exacerbating neuronal apoptosis following early brain injury [41].

The total number of altered lncRNAs tends to correlate with the severity of brain injury which suggests their role in the pathological mechanisms associated with TBI [9, 42, 43]. MALAT-1 was reported to control the key processes of angiogenesis following TBI by targeting the EZH2/NOTCH1 pathway [20]. In this study, MALAT-1 showed a significant increase in its expression levels in sTBI patients compared to the control and those with mTBI or modTBI. Furthermore, this increase in expression level escalated over time, reaching its peak after 30 days. On the contrary, MALAT-1 was increased 6 h after TBI in rodents and then reached the lowest level at day seven [20]. In our research, MALAT-1 emerged as a highly discriminative biomarker distinguishing between severe TBI, mild TBI, and moderate TBI at initial assessment, as well as at 5 and 30 days following the injury.

Several miRNAs show potential as promising biomarkers that can unveil the primary and secondary molecular alterations following TBI [44]. MiRNAs have a unique role in regulating protein expression and neural circuit creation [15]. Damaged neurons appear to release miRNAs into the extracellular environment through microvesicles [45]. Our findings revealed a noteworthy increase in hsa-miR-16-5p levels in cases of all TBI patients in comparison to the healthy volunteer group and therefore can be used as a good biomarker for diagnosis at initial assessment, as well as at 5 and 30 days following the injury. Interestingly, people with mTBI had higher levels of hsa-miR-16-5p compared to those with modTBI or sTBI and its level increased after 30 days. Previously, hsa-miR-16-5p was found to increase in mTBI to sTBI cases [44]. This observation aligns with the conclusions drawn by Redell et al. [46] who reported the elevation of miR-16 levels within the first day in patients with mTBI, while those with sTBI had the lowest levels.

The GCS score has been recognized as a reliable indicator of injury severity and the evolution of brain damage [47]. Nevertheless, recent studies have highlighted its subjectivity and vulnerability to complex clinical situations like sedation or shock [48]. Consequently, the quest for reliable biomarkers that offer objective and quantifiable measures for assessing injuries and predicting outcomes is crucial. Our results proved by ROC curve analysis that hsa-miR-16-5p, AKT3, and GSK-3β can be a reliable indicator for diagnosis. Moreover, the fact that the expression level of different biomarkers changed at other times makes them helpful in evaluating the period after post-trauma. They also can be used for evaluation of prognosis, follow-up, and detection of severity of TBI. It is noteworthy that these biomarkers exhibited substantial alterations across the three groups and demonstrated the ability to differentiate between levels of severity. In contrast to CT scans that revealed changes within the three groups and were unable to distinguish between the varying levels of severity. Moreover, the current results proved a significant correlation between MALAT-1, GSK-3β and GCS. Previously, the lncRNA GAS5 was negatively correlated with GCS [49].

Bioinformatics was used to prove the indulgence of MALAT-1/hsa-miR-16-5p/AKT3/GSK-3β in the pathogenesis of TBI and get more insight into the cellular roles and patterns of regulation. lncRNA-miRNA-mRNA interaction network was constructed. hsa-miR-16-5p target genes were investigated. They were involved in cadherin binding and Ubl conjugation. Previously, TIMP2 was reported to treat TBI by cadherin internalization in mice [50]. Moreover, Studies suggest that zinc might help protect brain cells’ neurons from damage after a head injury by reducing a cellular process called ubiquitin conjugation [51]. Our investigations also revealed the involvement of hsa-miR-16-5p’s genes in apoptotic, mTOR, Rap1, calcium, Ras, and Wnt signaling pathways which contribute to the overall pathology of TBI [52]. mTOR inhibition and activation of the Epac/Rap1 signaling pathway may improve recovery after TBI in mice models. These treatments showed significant improvement in both thinking and movement abilities [53]. RAS plays an important role in TBI and may contribute to permanent brain damage after TBI [54]. The Wnt pathway plays an important role in TBI, especially WNT3A which showed a positive effect in promoting neuronal regeneration in TBI [55, 56]. These results will potentially open a new avenue of therapeutic strategies for TBI.

The small sample size was a limitation in our study because of the slow enrollment of eligible patients during the research period. Nevertheless, the study leaned more towards being exploratory rather than confirmatory, yielding some notable outcomes. Consequently, we anticipate that a study with a larger sample size will validate and broaden our discoveries. Moreover, the prognostic and follow-up utility of these biomarkers should be further investigated.

## Conclusion

In conclusion, this study explored that AKT3, GSK-3β, and hsa-miR-16a-5p had good diagnostic value in TBI patients at initial assessment, as well as at 5 and 30 days following the injury. Moreover, MALAT-1 had good diagnostic value in sTBI compared to mTBI and modTBI patients, and its prognostic value extends to 30 days. GSK-3β was an excellent biomarker for detecting mTBI. MALAT-1 showed a significant negative correlation with GCS. In contrast, GSK-3β showed a significant positive correlation with GCS. Owing to the preliminary study design, our findings still need further validation with larger sample size.

## Data Availability

No datasets were generated or analysed during the current study.
